# Associations of vitamin D deficiency and vitamin D receptor (Cdx-2, Fok I, Bsm I and Taq I) polymorphisms with the risk of primary open-angle glaucoma

**DOI:** 10.1186/s12886-016-0289-y

**Published:** 2016-07-19

**Authors:** Yingjuan Lv, Qingbin Yao, Wenjiang Ma, Hua Liu, Jian Ji, Xiaorong Li

**Affiliations:** Department of Glaucoma, Tianjin Medical University Eye Hospital, Tianjin Medical University Eye Institute, The School of Optometry&Ophthalmology, No.251 Fu Kang Road, Nan kai District, Tianjin, 300384 China; Department of Histology and Embryology, Tianjin Medical University, No. 22 Qi Xiang Tai Road, He Ping District, Tianjin, 300070 China

**Keywords:** Vitamin D deficiency, Vitamin D receptor, Polymorphism, Primary open-angle glaucoma

## Abstract

**Background:**

Vitamin D deficiency and vitamin D receptor gene polymorphisms are known to be significantly associated with high myopia. Whether this genetic variant may impact primary open-angle glaucoma is largely unknown. This study investigated whether vitamin D receptor gene polymorphisms are altered in primary open-angle glaucoma subjects carrying the risk allele, and whether vitamin D deficiency is an important factor in the development of glaucoma.

**Methods:**

Seventy-three POAG patients and 71 age-matched controls from the Han population were enrolled. Serum levels of 1a, 25-Dihydroxyvitamin D3 were measured by enzyme-linked immunoabsorbent assay. Vitamin D receptor polymorphisms (Cdx-2, Fok I, Bsm I and Taq I) were analyzed using real-time polymerase-chain reaction high resolution melting analysis.

**Results:**

Serum levels of 1a, 25-Dihydroxyvitamin in primary open-angle glaucoma patients were lower than in age-matched controls. Statistical analysis revealed a significant difference in the allelic frequencies of the BsmI and TaqI genotypes between primary open-angle glaucoma patients and age-matched controls, while other polymorphisms did not show any significant differences.

**Conclusions:**

Vitamin D deficiency and the presence of the BsmI ‘B’ allele and the TaqI ‘t’ allele are relevant risk factors in the development of glaucoma.

**Trial registration:**

Clinical Trials.gov: NCT02539745.

The study was registered retrospectively on August 3rd, 2015. The first participant was enrolled on July 4th, 2013.

## Background

Glaucoma is characterized by typical structural damage to the optic nerve, specific visual field defects, and often relatively higher intraocular pressure (IOP) [1, 2]. It is also a complex inherited disorder for which an increasing number of genetic associations have been described, each contributing modestly to disease burden [3]. Primary open-angle glaucoma (POAG) is the most common type of glaucoma in all populations [4]. The molecular mechanisms leading to the pathogenesis of POAG are not completely understood. Genetic factors have been regarded as a critical risk factor in the pathogenesis of POAG [5]. Although the gene mutations in various populations have been identified by genetic studies and a genetic basis for POAG pathogenesis has been established [6–9], further identification of the genetic basis of glaucoma should help delineate the pathogenesis of the disease [10].

Vitamin D is now recognized as a versatile signaling molecule rather than being solely a regulator of bone health and calcium homeostasis [11]. It is involved in the modulation of different biological processes, including skeletal metabolism, immunological response, proliferation and differentiation of cells [12]. 1a, 25-Dihydroxyvitamin D3 is the endogenously produced, hormonally active form of vitamin D. 1a, 25-Dihydroxyvitamin D3 elicits its action on target tissues through the single vitamin D receptor (VDR) [13–18]. Recent studies have demonstrated an association between vitamin D levels and myopia; vitamin D deficiency has been shown to influence the development of high myopia [19]. Many clinical and research studies have shown that high myopia and glaucoma are closely associated and have proved that myopia was a risk factor for POAG [20–22]. Vitamin D levels are also associated with glaucoma. A recent article proposed that 1a, 25-Dihydroxyvitamin D3, or an analog thereof, may be used to treat glaucoma [23, 24]. Therefore, serum 25-Dihydroxyvitamin D3 levels may be of critical concern to POAG patients.

The vitamin D receptor gene (VDR) has been identified as a genetic factor that may contribute to spine pathologies [25, 26]. Several single nucleotide polymorphisms (SNPs) have been identified in the VDR sequence [13, 27]. The presence of VDR gene polymorphisms has been regarded as a critical risk factor in the development of ocular disease. For instance, FokI polymorphism has been associated with high myopia [13].

Few studies have shown an association between vitamin D deficiency and glaucoma, and there are no previous studies relating VDR gene polymorphisms to the development of glaucoma. In this study, we will evaluate whether vitamin D deficiency and Cdx-2, FokI, BsmI and TaqI polymorphisms of the VDR gene are associated with POAG in the Han population of China. The present study can provide a platform to help explain the pathological mechanism of POAG and demonstrate the geographic and ethnic differences which were associated with disease incidence and mortality.

## Methods

### Subjects

This was a hospital-based and case–control study. 71 POAG patients and 73 randomly selected age-matched controls from the Han population at Tianjin Medical University Eye Hospital were voluntarily enrolled. The group included 68 males and 76 females, with ages ranging from 55–65 years. All individuals underwent standardized clinical examinations for glaucoma at Tianjin Medical University Eye Hospital during 2013–2014. These examinations include slit-lamp biomicroscopy, gonioscopy, automated visual field testing (Octopus G1; Interzeag, Schlieren, Switzerland), fundus photography (Carl Zeiss Meditec, Oberkochen, Germany), optional laser scanning tomography (HRT II; Heidelberg Engineering, Heidelberg, Germany) of the disc and a 24-h Goldmann-applanation intraocular pressure (IOP) tonometry profile with six measurements [28]. All POAG patients met the following four inclusion criteria [29–32]: (1) intraocular pressure greater than 21 mmHg or more in each eye without therapy; (2) wide anterior chamber angle; (3) glaucomatous optic neuropathy (glaucomatous optic nerve damage was defined as cup-to-disc ratio higher than 0.7 or focal loss of the nerve fiber layer (notch) associated with a consistent glaucomatous visual field defect in at least one eye); (4) visual field loss consistent with optic nerve damage (visual fields were determined using standard automated perimetry in at least one eye). Exclusion criteria included the presence of any secondary glaucoma including exfoliation syndrome or a history of ocular trauma, high myopia, macular degeneration, other ocular diseases, a known history of systemic diseases, and administration of vitamin D3 or other analog. The controls were also checked for anterior chamber angle, fundus, and intraocular pressure, based on their past medical records and interviews. The controls were selected based on criteria which included: no family history of glaucoma or ocular hypertension; IOP less than 20 mmHg in both eyes in at least one of their last two checkups; CCT greater than 500 μm in both eyes; no visual field defect; cup discs that were physiologic and similar in both eyes; a cup-to-disc ratio <0.2; no defect in disc rim or margin; and no splinter hemorrhage around the disc. Individuals with high myopia, macular degeneration, hypertension, systemic diseases, family history of glaucoma, or a history of administration of vitamin D3 or its analog were excluded from the control group.

### Blood sample processing and biomarker assays

Fasting blood samples were drawn at 8 am from all study participants. Ethylenediaminetetraacetic acid (EDTA K2) blood-collection tubes were immediately stored on ice and then centrifuged within 30 min at 2,300 rpm for 15 min at 4°C. The separated plasma was immediately transferred to polypropylene tubes and stored at −20°C. Serum levels of 1a, 25-Dihydroxyvitamin D3 were measured by enzyme-linked immunoabsorbent assay, using commercially available kits (Trust Specialty Zeal biological trade Co., Ltd., U.S.A). 1a, 25-Dihydroxyvitamin D3 was measured in ng/ml, according to previously described methods [33].

Sodium citrate blood-collection tubes were stored at −20°C. Genomic DNA from the blood samples was extracted using a DNA Extraction Kit (TIANGEN) and stored at 4°C. DNA was screened for Cdx-2, FokI, BsmI and TaqI mutations of VDR gene [34] using high resolution melting analysis (PCR-HRM) on a Roche LightCycler480 platform. All the mutations detected in HRM were confirmed by sequencing. All the primers were as described before (Table 1). Cdx-2, FokI, BsmI and TaqI are reported according to the standard nomenclature in which lowercase and uppercase letters indicate the presence or absence of a restriction site, respectively. The FokI T and C alleles are represented by f and F, the BsmI G and A alleles by b and B, and the TaqI T and C alleles by t and T, respectively, and the Cdx-2 binding site polymorphism (G to A) in the promoter region of the human vitamin D receptor gene alleles by G and A [35, 36].

**Table 1:** Primers for VDR gene polymorphisms (Cdx-2,FokI,BsmI and TaqI) (GenBank AY342401)

SNP	Primer(5’ → 3’)	Primer(5’ → 3’)	bp
Cdx-2	GGGTCTTCCCAGGACAGTAT	GGAATGAAAGAGGGAAGGAG	191
FokI	GTCAGGCAGGGAAGTGCTG	CTGGCACTGACTCTGGCTC	86
BsmI	GCAAGAAACCTCAAATAACAGG	ATTCTGAGGAACTAGATAAGCAGG	121
TaqI	TACGTCTGCAGTGTGTTGGA	CTGAGAGCTCCTGTGCCTTC	115

### Statistical analysis

Age comparison between POAG patients and age-matched controls was analyzed using Student’s *t* test. Gender comparison between the two groups was analyzed using a chi-square test. A power analysis using PASS software was used to assess the sample size. Statistical differences of serum levels of 1a, 25-Dihydroxyvitamin D3 between the two groups were evaluated by Student’s *t* test. Statistical differences of genotype and allele frequencies of patients with POAG were evaluated by chi-square test and logistic regression analysis. The odds ratio (OR) and 95 % confidence interval (CI) were calculated to assess the relative risk of POAG in relation to a specific allele [28]. Statistical analysis used SPSS17.0 software and *p* < 0.05 was considered statistically significant.

## Results

Age and gender comparisons between POAG patients and age-matched controls were not statistically significant (*p*>0.05) (Tables 2, and 3). Sample size analysis using PASS software produced a power of 0.998 in this study.

**Table 2:** Age comparison between POAG patients and age-matched people

Group	*n*	age		*t* value	*P* value
		Mean	SD		
Control	73	60.14	3.03	1.844	0.067
POAG	71	61.03	2.75

**Table 3:** Gender comparison between POAG patients and age-matched people

Group	*n*	male	female	*X* ^2^	*P* value
Control	73	29	44	3.338	0.068
POAG	71	39	32		

### Analysis of serum levels of 1a, 25-Dihydroxyvitamin D3

Serum levels of 1a, 25-Dihydroxyvitamin D3 were measured for both 71 POAG patients and 73 age-matched controls. The mean ± SD serum levels of 1 a, 25-Dihydroxyvitamin D3 were 30.43 ± 3.91 ng/ml in age-matched controls and 26.37 ± 5.83 ng/ml in POAG patients. The serum levels of 1 a, 25-Dihydroxyvitamin D3 in age-matched controls was significantly higher than the levels in POAG patients. (*p* < 0.001) (Table 4).

**Table 4:** Serum levels of 1a, 25-Dihydroxyvitamin D3 comparing POAG patients with age-matched people

Group	*n*	1a, 25-Dihydroxyvitamin D3		*t* value	*P* value
		Mean	SD		
Control	73	30.43	3.91	4.920	<0.001
POAG	71	26.37	5.83		

### Analysis of allele and genotype frequencies of VDR gene polymorphism with respect to POAG (Table 5)

**Table 5:** Genotype distributions and allelic genes of VDR gene (Cdx-2,FokI,BsmI,TaqI) polymorphisms comparing POAG patients with age-matched people

VDR genetype	Control	POAG	*X* ^2^	*P* value	OR(95%CI)
	(*n* = 73)	(*n* = 71)			
Cdx-2					
GG	26	31	0.978	0.613	
AG	32	27			
AA	15	13			
G	84	89	0.632	0.427	1.239 (0.773 ~ 1.988)
A	62	53			
FokI					
FF	19	19	3.278	0.194	
Ff	27	35			
ff	27	17			
F	65	73	1.368	0.242	1.318 (0.829 ~ 2.096)
f	81	69			
BsmI					
Bb	4	18	10.982	0.001	5.858 (1.872 ~ 18.337)
bb	69	53			
B	4	18	10.074	0.002	5.153 (1.699 ~ 15.635)
b	142	124			
TaqI					
TT	66	53	6.234	0.013	
Tt	7	18			3.202 (1.245 ~ 8.238)
T	139	124	5.641	0.018	
t	7	18			2.882 (1.165 ~ 7.132)

**Fig. 1:**
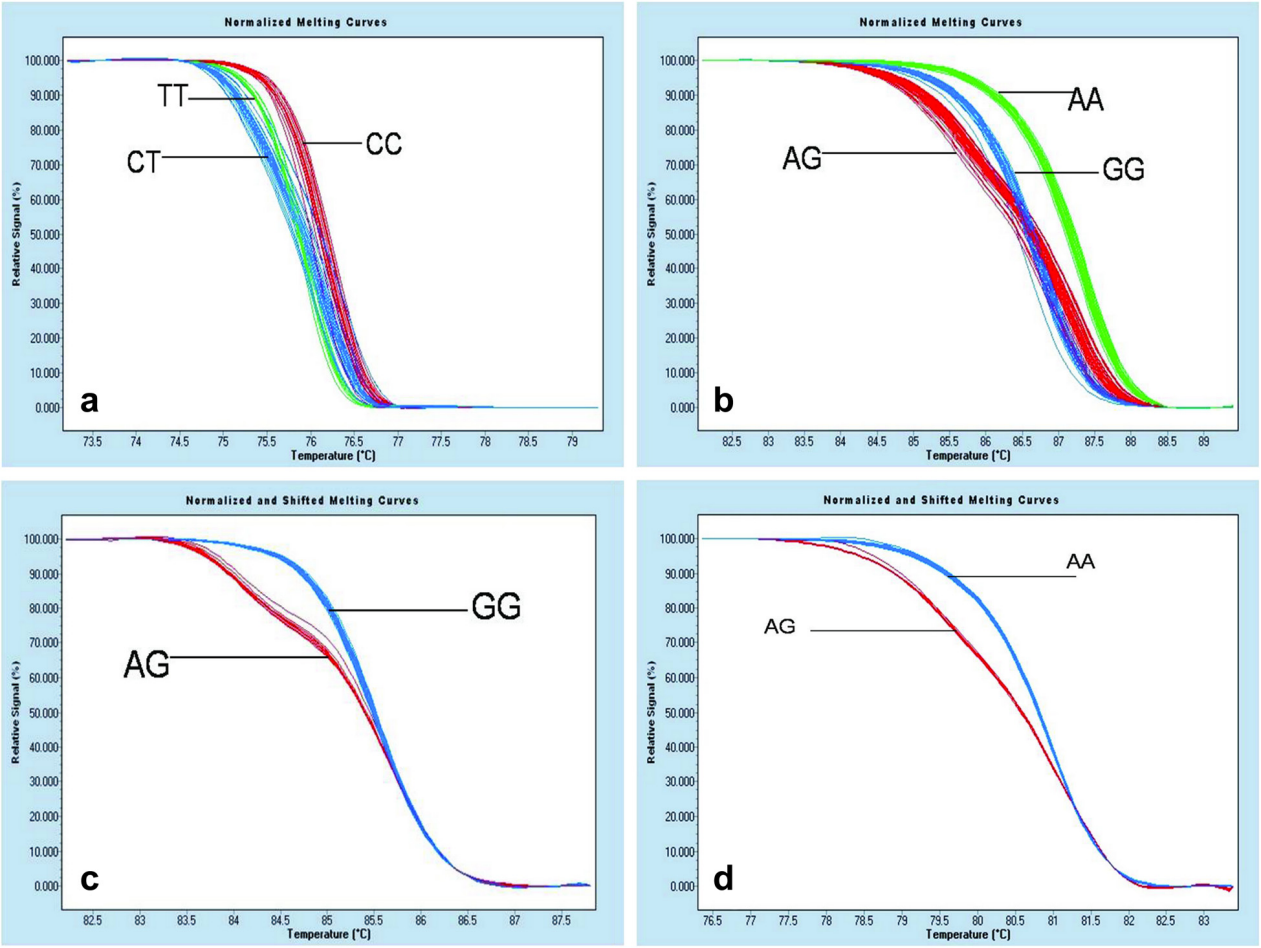
Genotypes of VDR gene (**a**: Cdx-2, **b**: Fok I, **c**:Bsm I, **d**: Taq I) polymorphisms were determined by PCR-HRM

VDR polymorphic analysis (Cdx-2, Fok I, Bsm I and Taq I) was performed using PCR-HRM (Fig. 1 a: Cdx-2, b: Fok I, c:Bsm I, d: Taq I).

The genotype distribution of the Cdx-2 polymorphism in POAG patients revealed a considerable increase in the frequency of the GG homozygote (43.66 %) compared to the control group (35.62 %), a decrease in the frequency of the AA homozygote (18.31 %) compared to the control group (20.56 %), and a corresponding decrease in the frequency of AG heterozygote (38.03 % in POAG vs. 43.83 % in controls) without much variation in heterozygote frequencies (*P* =0.613; *χ*2 = 0.978). The allelic distribution revealed reduction in the G allele frequency (GG and AG) in POAG patients (62.68 %) compared to the control group (57.53 %) (*p* = 0.427; *χ*2 = 0.632; OR = 1.239, 95 % CI: 0.773 ~ 1.998).

The genotype distribution of the Fok1 polymorphism in POAG patients revealed a considerable increase in the frequency of the FF homozygote (26.76 %) compared to the control group (26.02 %), an increase in the frequency of the Ff heterozygote (49.30 %) compared to the control group (36.99 %), and a corresponding decrease in the frequency of the ff homozygote (23.94 % in POAG vs. 36.99 % in controls) without much variation in heterozygote frequencies (*p* = 0.194; *χ*2 = 3.278). The allelic distribution revealed an increase in the F allele frequency (FF and Ff) in POAG patients (51.41 %) as compared to that of control group (44.52 %) (*p* = 0.242; *χ*2 = 1.368; OR = 1.318, 95 % CI:0.829 ~ 2.096).

The genotype distribution of the BsmI polymorphism in POAG patients revealed a considerable increase in the frequency of the Bb heterozygote (25.35 %) compared to the control group (5.48 %), and a corresponding decrease in the frequency of the bb homozygote (74.65 % in POAG vs. 94.52 % in controls) with much variation in heterozygote frequencies (*p* = 0.001; *χ*2 = 10.982). The allelic distribution revealed reduction in the B allele frequency (Bb) in POAG patients (12.68 %) compared to the control group (2.74 %) (*p* = 0.002; *χ*2 = 10.074; OR = 5.153, 95 % CI: 1.699 ~ 15.635).

The genotype distribution of the Taq I polymorphism in POAG patients revealed a considerable decrease in the frequency of the TT homozygote (74.65 %) compared to the control group (90.41 %), with a corresponding increase in the frequency of the Tt heterozygote (23.35 % in POAG vs. 9.59 % in controls) and much variation in heterozygote frequencies (*p* = 0.013; *χ*2 = 6.234). The allelic distribution revealed an increase in the t allele frequency (Tt) in POAG patients (12.68 %) compared to the control group (4.79 %) (*p* = 0.018; *χ*2 = 5.641; OR = 2.882, 95 % CI:1.165 ~ 7.132).

## Discussion

### Vitamin D deficiency may affect the incidence and progression of POAG

Vitamin D is an inactive precursor when produced in the skin utilizing the energy of sunlight or when ingested as a dietary vitamin D [11, 12]. It requires two hydroxylation steps: first in the liver and then in the kidney [11]. It can be converted to 1α, 25-dihydroxyvitamin D3, which is the active hormone [11, 17, 18]. Levels of 1a, 25-Dihydroxyvitamin D3 are controlled by numerous factors. In low-calcium states, levels of 1a, 25-Dihydroxyvitamin D3 increase due to parathyroid hormone activity increases. However, in serious low-calcium states, when the substrate is exhausted, levels of 1a, 25-Dihydroxyvitamin D3 decrease [17, 18, 37]. In this study, serum levels of 1a, 25-Dihydroxyvitamin D3 in POAG patients were significantly lower than age-matched controls. These results suggest that Vitamin D deficiency might contribute to an increase in the incidence of POAG and may play an important role in the development of POAG. In POAG patients, the cause of 1a, 25-Dihydroxyvitamin D3 deficiency is unclear. However, topical administration of 1a, 25-dihydroxyvitamin D3 markedly reduces IOP in non-human primates [23]. The exact mechanism by which 1a, 25-(OH)2D3 reduces IOP is also unclear.

### The BsmI ‘B’ allele and the TaqI ‘t’ allele may affect the development pathway of POAG

1a, 25-dihydroxyvitamin D3 regulates genes that are known to be involved in the determination of intraocular pressure (IOP) [23]. Moreover, ocular hypertension is the greatest known risk factor for POAG. The currently accepted mechanism is that vitamin D implements its functions through a VDR [17–20], which is present in most cell types in tissues. When 1α, 25-dihydroxyvitamin D3 binds to the VDR, it forms 1α, 25-dihydroxyvitamin D3/VDR complexes, regulating multiple target genes in tissues containing the VDR [21, 22]. The VDR not only regulates transcriptional responses but is also involved in micro RNA-directed post-transcriptional mechanisms [25, 26]. In humans, VDR is involved in retinoid functions of the eye such as accommodation, pupil responses, and aqueous humor production, and may have an indirect and functional role in ocular growth and myopia development [13, 23].

The VDR gene encodes the VDR. Several single nucleotide polymorphisms (SNPs) have been identified in the VDR gene, which truncate the VDR protein, such as FokI at the 5’ end of exon 2 (rs10735810), Cdx-2 binding site locus upstream of exon 1e (rs11568820), BsmI in intron 8 (rs1544410) and TaqI in exon 9 (rs731236) in the 3’UTR region [27, 33, 34]. In this study, each SNP demonstrated the Hardy-Weinberg equilibrium. Statistical analysis revealed a significant difference in the allelic frequencies of the BsmI and TaqI genotypes, while other polymorphisms did not show any significant differences. The protective actions of the BsmI ‘B’ allele and the Taq I ‘t’ allele were confirmed experimentally, leading to a better understanding of the pathological mechanism of glaucoma. Firstly, VDR gene polymorphisms can regulate the metabolism of vitamin D by positive and negative feedback, including the metabolism of 1a, 25-Dihydroxyvitamin D3. Secondly, the BsmI and TaqI in the 3’UTR region may be relevant to VDR mRNA stability and gene transcription, which is involved in the following: production and outflow of aqueous humor, remodeling of the extracellular matrix in the trabecular meshwork, controlling intraocular pressure and participating in the development of POAG. Therefore, the BsmI ‘B’allele and the TaqI ‘t’ allele may be important risk factors in the development of glaucoma.

## Conclusions

Vitamin D deficiency, the BsmI ‘B’allele and the TaqI ‘t’ allele point to their direct roles in POAG development. However, the causes of 1a, 25-Dihydroxyvitamin D3 deficiency, the changes in the structure and function of VDR and the frequency of allele carriers of polymorphisms of VDR require further study. The possibility of administering vitamin D3 to POAG patients who have low levels of 1a, 25-Dihydroxyvitamin D3, and the question of whether 1a, 25-Dihydroxyvitamin D3 affects intraocular pressure should be further investigated.

## Abbreviations

IOP, intraocular pressure; POAG, primary open-angle glaucoma; PAC, primary angle closure; EDTA K2, ethylenediaminetetraacetic acid; SNPs, single nucleotide polymorphisms; OR, odds ratio; CI, confidence interval; VDR, vitamin D receptor; SD, standard deviation; n, number
